# TrCla4 promotes actin polymerization at the hyphal tip and mycelial growth in *Trichophyton rubrum*

**DOI:** 10.1128/spectrum.02923-23

**Published:** 2023-10-31

**Authors:** Masaki Ishii, Yasuhiko Matsumoto, Tsuyoshi Yamada, Hideko Uga, Toshiaki Katada, Shinya Ohata

**Affiliations:** 1 Research Institute of Pharmaceutical Sciences, Faculty of Pharmacy, Musashino University, Nishitokyo-shi, Tokyo, Japan; 2 Department of Microbiology, Meiji Pharmaceutical University, Kiyose, Tokyo, Japan; 3 Teikyo University Institute of Medical Mycology, Teikyo University, Hachioji, Tokyo, Japan; 4 Asia International Institute of Infectious Disease Control, Teikyo University, Hachioji, Tokyo, Japan; CNRS-Inserm-Université Côte d'Azur, Nice, France

**Keywords:** dermatophytosis, p21-activated kinase, PAK inhibitor, IPA-3, actin polymerization, *Trichophyton rubrum*

## Abstract

Dermatophytes invade and colonize host superficial tissues via hyphal growth. Although cytoskeletal reorganization and its regulation are essential for hyphal growth, the molecular mechanisms in dermatophytes and their applicability as antifungal drug targets remain poorly understood. The p21-activated kinase (PAK) is a downstream effector of the small GTPases Rac and CDC42, also known as p21, and is involved in various molecular and cellular functions, including actin polymerization and cell morphogenesis. In this study, we investigated the contribution of the PAK protein TrCla4 to morphogenesis and mycelial growth in *Trichophyton rubrum*, the most frequently isolated fungus in dermatophytosis (athlete’s foot). The actin polymerization inhibitor, cytochalasin A inhibited actin accumulation at the hyphal tip and mycelial growth of *T. rubrum*, suggesting the involvement of the actin cytoskeleton in mycelial growth. In the Tr*cla4* knockout strain (ΔTr*cla4*), we observed defects in mycelial growth, hyphal branching, and the accumulation of polymerized actin at the hyphal tip. Chemical inhibitors of TrRac-dependent TrCla4 kinase activity, FRAX486 and IPA-3, also inhibited spore germination and mycelial growth. Interestingly, ΔTr*cla4* showed no additional inhibition of mycelial growth when treated with these inhibitors, indicating that their inhibitory effects are primarily mediated through TrCla4. In an invertebrate dermatophyte infection model, animals infected with ΔTr*cla4* had higher survival rates than those infected with the wild-type, and IPA-3 and FRAX486 treatments both significantly improved animal survival rates. These results suggest that the dermatophyte PAK promotes mycelial growth by facilitating actin polymerization at the hyphal tip, making it a potential therapeutic target for dermatophytosis.

**IMPORTANCE:**

Superficial fungal infections, such as athlete’s foot, affect more than 10% of the world’s population and have a significant impact on quality of life. Despite the fact that treatment-resistant fungi are a concern, there are just a few antifungal drug targets accessible, as opposed to the wide range of therapeutic targets found in bacterial infections. As a result, additional alternatives are sought. In this study, we generated a PAK TrCla4 deletion strain (∆Trcla4) of *Trichophyton rubrum*. The ∆Trcla4 strain exhibited deficiencies in mycelial growth, hyphal morphology, and polarized actin localization at the hyphal tip. IPA-3 and FRAX486, small chemical inhibitors of mammalian PAK, were discovered to limit fungal mycelial proliferation. According to our findings, fungal PAKs are interesting therapeutic targets for the development of new antifungal medicines.

## INTRODUCTION

Dermatophytes are the most frequent fungus that causes superficial mycosis, which affects 20–25% of the world’s population (1). Itching, deterioration of the look of the toes and nails, and trouble walking owing to nail abnormalities are all symptoms of the illness. Furthermore, it has been linked to an increase in asthma exacerbations (2, 3). Dermatophytosis is treated with azole and arylamine antifungal drugs, which are biosynthesis inhibitors of ergosterol, an important fungal sterol. Resistance to these medications has been documented in recent years (4, 5). Resistance to one azole medicine, itraconazole, is cross-resistant to voriconazole, another azole drug, in these resistant strains (4). This result highlights the possibility of treatment refractoriness by fungi that have developed poor sensitivity to broad-spectrum medicines acting on the same target molecules. Antifungal medications’ target molecules are restricted, and there is a need to find new targets and produce treatments that target these molecules.

The most often identified dermatophytosis pathogen, *Trichophyton rubrum*, invades its host’s epidermal tissues through hypha, a filamentous form of the fungal cells (6 – 8). The proliferation of hyphal fragments or spore germination is followed by hyphal formation. The polar expansion of hyphae requires directed vesicular transport toward the hyphal tip, which provides lipids and proteins for the cell surface layer and the equipment for synthesizing the cell wall (9). In model filamentous fungi *Aspergillus nidulans* and *Neurospora crassa*, actin polymerization inhibition by cytochalasin A inhibits mycelial development and results in aberrant hyphal tip shape by delocalizing actin and vesicle-associated membrane proteins at the hyphal tip, accumulating extracellular secretory proteins intracellularly, and ectopically accumulating cell walls (10 – 12). These results imply that the polar development of the fungal cells is related to the polymerization of actin at the hyphal tip. Despite the fact that it is believed that polarized actin localization and polymerization at the hyphal tip are crucial for mycelial development in dermatophytes as well, its role in mycelial growth and the mechanism governing it are not well known.

Rac and CDC42, commonly known as p21, belong to the Rho family of small GTPases that control actin dynamics (13, 14). Rac and CDC42 are transformed from the inactive guanosine diphosphate (GDP)-bound form to the activated guanosine triphosphate (GTP)-bound form upon signaling and bind to their effector molecules to govern numerous biological processes such as cell polarization and cell morphogenesis (15). The p21-activated kinase (PAK) family of protein kinases are Rac/CDC42 effectors that engage with activated Rac/CDC42 through the CDC42/Rac-interactive binding (CRIB) motif at the N-terminus (15, 16). The activated PAK then phosphorylates its target molecules through its C-terminal kinase domain. In yeast, type-I myosins, which are necessary for actin polymerization, are phosphorylated *in vitro* by the yeast PAKs Cla4 and Ste20, and type-I myosins phosphorylation at serine 357 is required for actin assembly (17, 18). In yeast, PAKs also phosphorylated and controlled additional cytoskeleton regulators (a guanine nucleotide exchange factor of p21, Cdc24, and septins, Cdc10, and Cdc3) (19, 20). Compounds that block Rac/CDC42-PAK signaling are predicted to be useful not just as diagnostic tools for fungal signaling pathways but also as novel antifungal medicines. However, the role of PAK in *T. rubrum* and the efficacy of fungal PAK inhibitors in the treatment of fungal infections remain unknown.

In the present study, we found that TrCla4, a PAK in dermatophytes, promotes mycelial growth possibly via actin polymerization and that low-molecular-weight inhibitors targeting TrCla4, namely IPA-3 and FRAX486, interfere with the mycelial growth of the fungus and improve infection pathogenesis in an invertebrate infection model. Our study suggests that inhibition of mycelial growth by TrCla4 inhibitors may be a novel therapeutic strategy against dermatophytosis.

## RESULTS

### Rac/CDC42-PAK interaction inhibitor IPA-3 inhibits germination and mycelial growth in *T. rubrum*

**Fig 1:**
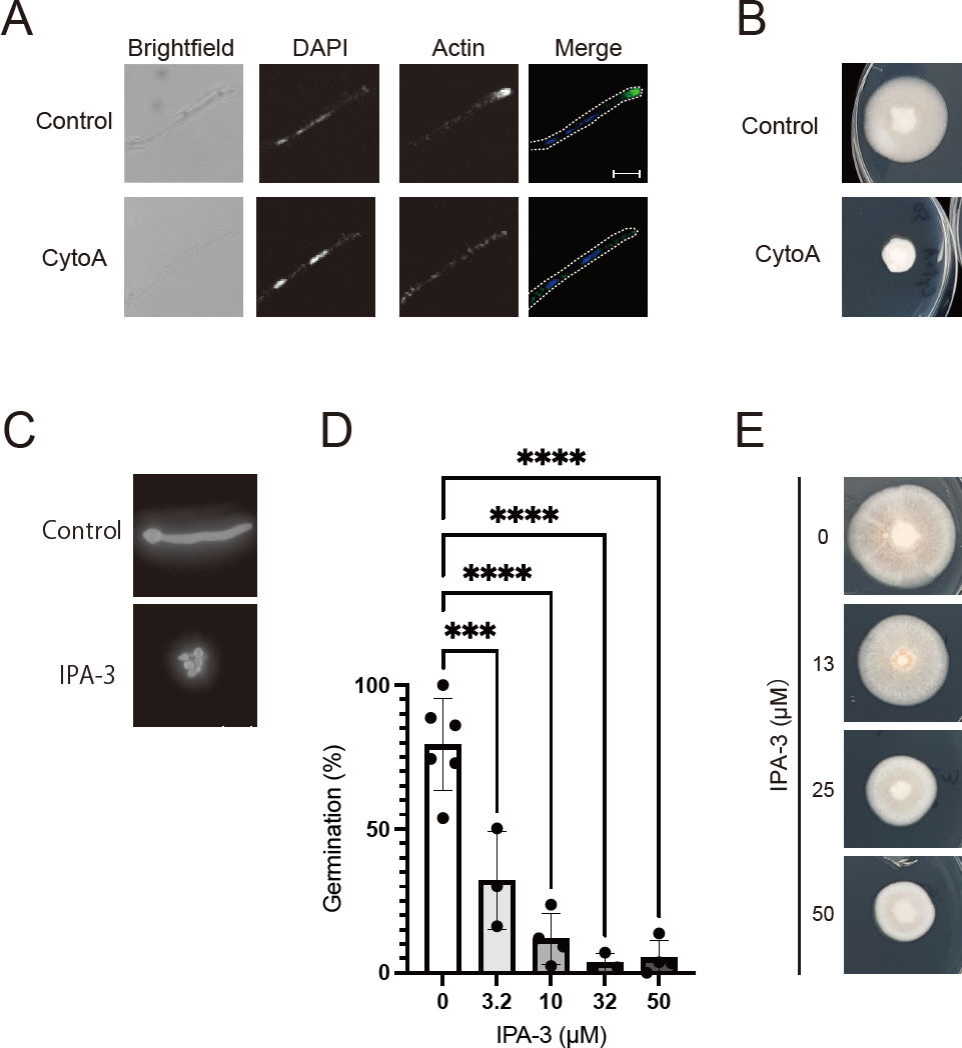
Effect of cytochalasin A on actin localization at the hyphal tip and mycelial growth in *T. rubrum*. (A) *T. rubrum* spores were cultured in SD medium on coverslips at 28°C for 2 days, followed by medium exchange with or without 16 µM cytochalasin A (CytoA) and incubation for 1 h. Hyphae were fixed with 4% PFA and stained with DAPI and anti-beta-actin antibody (C-4), followed by visualization of nuclei and beta-actin using Alexa 488-conjugated secondary antibodies. Scale bar: 10 µm. (B) *T. rubrum* spores were incubated on SDA plates with or without 16 µM cytochalasin A (CytoA) for 10 days. (C) Germination of *T. rubrum* spores treated with or without 32 µM of the p21-PAK interaction inhibitor IPA-3 for 21 h was observed using fluorescent microscopy. Spores were stained with Calcofluor white. Scale bar: 10 µm. (D) The germination ratio of *T. rubrum* spores treated with 0, 3.2, 10, 32, or 50 µM of the IPA-3 (*n* = 6, 3, 4, 3, and 4, respectively). Germinated and ungerminated spores were counted, and the ratio of germinated spores was calculated. The dots on the graph represent the germination ratio of individual samples. (E) The mycelial growth of *T. rubrum* incubated on SD agar with 0, 13, 25, or 50 µM of the IPA-3 for 10 days is shown.

To visualize the subcellular localization of actin in *T. rubrum*, we first performed phalloidin staining, which visualizes F-actin in higher eukaryotes (21, 22). However, we did not obtain a specific signal at the hyphal tip in *T. rubrum* (Fig. S1), as has been reported in other fungi (10 – 12). We therefore used anti-actin immunostaining. Consistent with findings in *A. nidulans* and *N. crassa* (10 – 12), actin was accumulated at the tips of hyphae and this accumulation was disrupted by treatment with cytochalasin A (Cyto A), an inhibitor of actin polymerization (Fig. 1A; Fig. S2A). Furthermore, Cyto A inhibited the growth of *T. rubrum* mycelia (Fig. 1B). Based on these findings, which suggest the significance of actin polymerization in the mycelial growth of *T. rubrum*, we focused our investigation on the Rac/CDC42-PAK pathway known to promote actin polymerization (23). IPA-3, an inhibitor that blocks the interaction between Rac/CDC42 and PAKs, has been shown to inhibit the activation of mammalian class 1 PAKs (24). To explore the potential involvement of the Rac/CDC42-PAK pathway in spore germination and mycelial growth in *T. rubrum*, we treated *T. rubrum* spores with IPA-3. The results demonstrated a dose-dependent inhibition of spore germination, with an IC_50_ value of 1.5 µM (Fig. 1C and D). Additionally, IPA-3 suppressed mycelial growth on SDA plates (Fig. 1E). These findings suggest that IPA-3 hinders the interaction between Rac/CDC42 and PAK in *T. rubrum*, and that the Rac/CDC42-PAK pathway plays a crucial role in spore germination and mycelial growth in *T. rubrum*.

**Fig 2:**
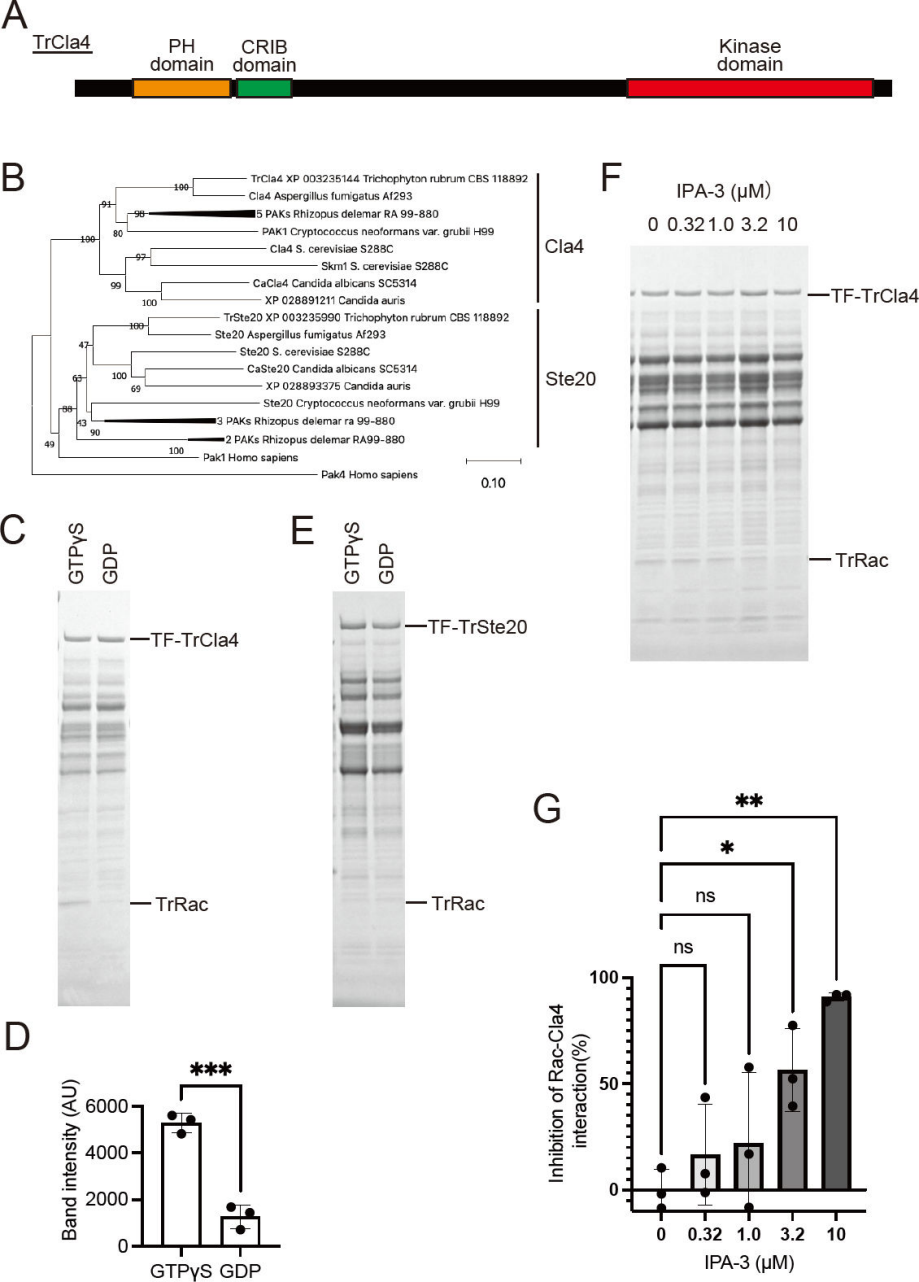
Interaction of *T. rubrum* PAK TrCla4 with p21 TrRac and its inhibition by IPA-3. (A) Schematic diagram illustrating TrCla4. (B) Phylogenetic tree of fungal PAKs inferred using the neighbor-joining method. The optimal tree is displayed. Evolutionary distances were calculated using the Poisson correction method, represented in units of amino acid substitutions per site. *Rhizopus delemar* RA 99–880, which possesses five *cla4*-like genes and five *ste20*-like genes, is depicted on one or two branches. (C and D) Ni NTA agarose beads and lysate of *E. coli* BL21 expressing Tf-TrCla4 were incubated with GTPγS or GDP-bound TrRac at 4°C for 2 h. The eluates were electrophoresed and stained with CBB (C). Left lane: pull-down sample with GTPγS-loaded TrRac; right lane: sample with GDP-loaded TrRac. The band intensity of pulled-down TrRac was measured (D). The data shown represent the mean ± standard deviation (SD), *n* = 3 each. The dots on the graph represent the band intensity of individual samples. (E) Ni NTA agarose beads and lysate of *E. coli* BL21 expressing Tf-TrSte20 were incubated with GTPγS or GDP-bound TrRac at 4°C for 2 h. The eluates were electrophoresed and stained with CBB. Left lane: pull-down sample with GTPγS-loaded TrRac; right lane: sample with GDP-loaded TrRac. (F and G) Ni NTA agarose beads and lysate of *E. coli* BL21 expressing Tf-TrCla4 were incubated with GTPγS-bound TrRac in the presence of DMSO (Control) or 0, 0.32, 1.0, 3.2, and 10 µM IPA-3 at 4°C for 2 h. The eluates were electrophoresed and stained with CBB (F). The band intensity of TrRac was measured (G). The data shown represent the mean ± standard deviation (SD), *n* = 3 each.

To identify PAKs in *T. rubrum*, we conducted a database search using the CRIB domain of human Pak1, which is responsible for PAKs’ interaction with Rac/CDC42. Through this search, we identified two candidate genes for PAK homologs (XP_003235144 and XP_003235990) in the *T. rubrum* CBS118892 genome. Both gene products possess CRIB and kinase domains, while only XP_003235144 contains a Pleckstrin homology (PH) domain. The PH domain is involved in interactions with phosphoinositol 4-phosphate and localization to the polarized growth tip in budding yeast (Fig. 2A) (25). Based on their amino acid sequence similarity and domain composition compared to other fungal PAK homologs (Fig. 2B), we named XP_003235144 and XP_003235990 Tr*cla4* and Tr*ste20*, respectively. To investigate whether TrCla4 and TrSte20 can bind to active TrRac in its GTP-bound form, we performed a pull-down assay using TrRac and His- and Tf-tagged TrCla4 and TrSte20 (Tf-TrCla4 and Tf-TrSte20, respectively). When Tf-TrCla4 was used as the bait, the intensity of the band corresponding to TrRac bound to GTPγS (the active form) was significantly higher than that of GDP-bound inactive TrRac (Fig. 2C and D). However, when Tf-TrSte20 was used as the bait, the band intensity of GTPγS-bound TrRac was weak and comparable to that of GDP-bound TrRac (Fig. 2E). These results indicate that TrCla4, but not Tr*Ste20*, can interact with TrRac in a manner dependent on the type of guanine nucleotide bound to TrRac. Therefore, we focused on TrCla4 for subsequent analyses. To assess whether IPA-3 disrupts the interaction between TrRac and TrCla4, we added IPA-3 to the pull-down reaction. The interaction between Tf-Cla4 and GTPγS-bound TrRac was inhibited by IPA-3 (Fig. 2F and G), with an IC_50_ value of 2.3 µM. These findings suggest that IPA-3 interferes with the interaction between TrRac and TrCla4, indicating the involvement of the TrRac-TrCla4 signaling pathway in mycelial growth in *T. rubrum*.

### Ablation of Tr*cla4* results in aberrant mycelial growth and hyphal morphogenesis

**Fig 3:**
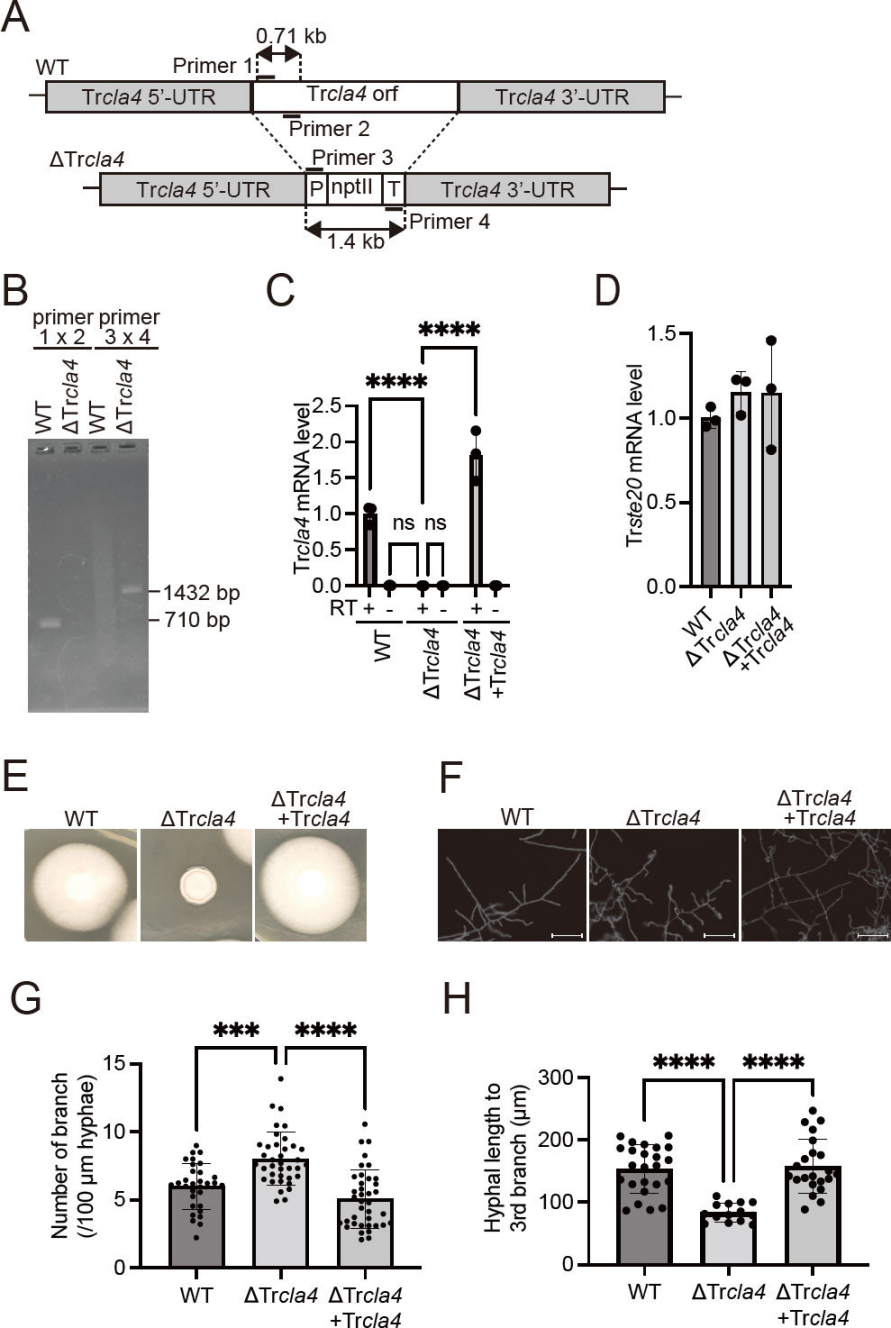
Contribution of the Tr*cla4* gene to mycelial growth and hyphal morphology in *T. rubrum*. (A) Schematic representation of the Tr*cla4* deletion construct. (B) PCR analysis of WT and ΔTr*cla4*. Genomic DNA samples were subjected to PCR using primer pairs 1 and 2 (lanes 1 and 2) or primer pairs 3 and 4 (lanes 3 and 4). Lanes 1 and 3 show amplified products from WT genome, while lanes 2 and 4 show DNA samples from ΔTr*cla4* genome. (C and D) Relative expression levels of Tr*cla4* (C) and Tr*ste20* (D) mRNAs in WT, ΔTr*cla4*, and ΔTr*cla4* + Tr*cla4* cultures after 1 week in SD medium. *rpb2* was used as an internal control. RT+ indicates reverse transcription, while RT− indicates no reverse transcription. The data shown represent the mean ± SD. Each point on the graph represents the relative mRNA expression level of an individual sample. *n* = 3 each. *P < 0.05. (E) Spores of WT, ΔTr*cla4*, and ΔTr*cla4 +* Tr*cla4* were inoculated on SDA for 10 days. (F) Spores of WT, ΔTr*cla4*, and ΔTr*cla4 +* Tr*cla4* were grown in SD medium with a coverslip at 28°C for 48 h. Hyphae were fixed with 4% PFA and stained with CFW to visualize cell walls. Bar represents 50 µm. (G) The number of branches per 100 µm length of hyphae was calculated. Each dot in the graph represents the number of branches in individual hyphae. *n* = 31 hypha from WT, *n* = 37 hypha ΔTr*cla4*, and *n* = 38 hypha from ΔTr*cla4 +* Tr*cla4*. (H) Hyphal length to the third branch was calculated. Each dot in the graph represents the number of branches in individual hyphae. *n* = 24 hypha from WT, *n* = 14 hypha from ΔTr*cla4*, and *n* = 22 hypha from ΔTr*cla4 +* Tr*cla4*.

To confirm the contribution of the TrRac-TrCla4 pathway to mycelial growth and hyphal formation, we generated a Tr*cla4* knockout mutant strain (ΔTr*cla4*) using *Agrobacterium*-mediated transformation (Fig. 3A). Ablation of Tr*cla4* was confirmed by PCR using primers designed within the ORF of Tr*cla4* (Primers 1 and 2), the promotor of *trpC* (P, Primer 3), and the terminator of *cgrA* (T, Primer 4; Fig. 3B). We also generated a strain in which Tr*cla4* was reintroduced into ΔTr*cla4* (hereafter referred to as ΔTr*cla4* + Tr*cla4*). Tr*cla4* mRNA expression level in ΔT*rcla4* was significantly lower than that in WT and comparable to the no-reverse transcription sample in WT (Fig. 3C). In ΔTr*cla4* + Tr*cla4*, Tr*cla4* mRNA level was significantly reconstituted (Fig. 3C). Although it was anticipated that the expression level of Tr*ste20*, the other possible PAK homolog, might be affected in ΔTr*cla4* and ΔTr*cla4* + Tr*cla4* to compensate for the change in the expression level of Tr*cla4*, the expression of Trste20 mRNA in ΔTr*cla4* and ΔTr*cla4* + Tr*cla4* was comparable to that of WT (Fig. 3D). These results suggest that ΔTr*cla4* and ΔTr*cla4* + Tr*cla4* are useful tools to analyze the function of Tr*cla4* in *T. rubrum*.

To gain insight into the role of Tr*cla4* in mycelial growth of *T. rubrum*, we cultured WT, ΔTr*cla4*, and ΔTr*cla4* + Tr*cla4* strains on SDA plates. As anticipated, ΔTr*cla4* exhibited reduced mycelial growth compared to WT and ΔTr*cla4* + Tr*cla4* (Fig. 3E), confirming the findings obtained with IPA-3 treatment that TrCla4 promotes mycelial growth (Fig. 1E). Microscopic examination revealed highly branched hyphae and shortened hyphal length in ΔTr*cla4* (Fig. 3F through H). These results provide evidence that TrCla4 plays a crucial role in promoting mycelial growth and maintaining the normal shape of hyphae in *T. rubrum*.

### Regulation of actin accumulation by TrCla4

**Fig 4:**
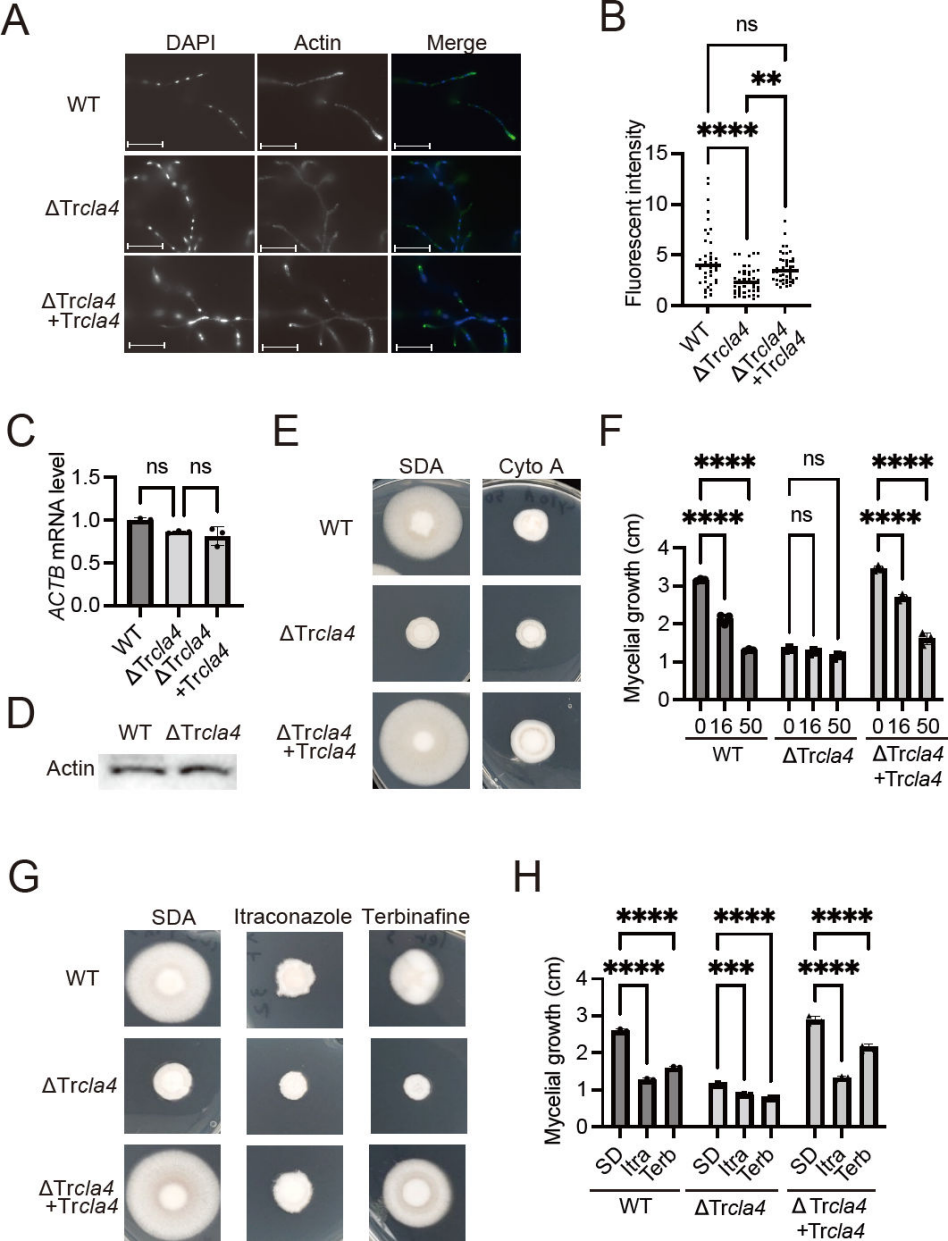
Effect of Tr*cla4* deletion on actin localization and sensitivity to the actin inhibitor cytochalasin A. (A) Spores of WT, ΔTr*cla4*, and ΔTr*cla4 +* Tr*cla4* were grown in SD medium with a coverslip at 28°C for 48 h. Hyphae were fixed with 4% PFA and stained with DAPI and anti-beta-actin antibody (C-4), followed by Alexa 488-conjugated secondary antibody to visualize nuclei and beta-actin, respectively. Bar represents 20 µm. (B) Fluorescent intensity of actin localized at the hyphal tip was calculated. Each dot in the graph represents the fluorescent intensity of an individual hyphal tip. *n* = 39 hyphal tip from WT, *n* = 44 hyphal tip from ΔTr*cla4*, and *n* = 49 hyphal tip from ΔTr*cla4 +* Tr*cla4*. (C) Relative expression levels of *ACTB* mRNAs in WT, ΔTr*cla4*, and ΔTr*cla4 +* Tr*cla4* cultures after 1 week in SD medium. *rpb2* was used as an internal control. The data shown represent the mean ± SD. Each point on the graph represents the relative mRNA expression level of an individual sample. *n* = 3 each. ns: not significant. (D) Western blot analysis of actin protein levels in WT and ΔTr*cla4* samples. The total protein load for both samples is 25 µg/lane. (E and F) Spores of WT, ΔTr*cla4*, and ΔTr*cla4 +* Tr*cla4* were incubated on an SDA plate with or without 50 µM cytochalasin A for 10 days (E). The diameter of the mycelium was measured (F). The dots on the graph represent the diameter of individual samples. *n* = 3 each. (G and H) Spores of WT, ΔTr*cla4*, and ΔTr*cla4 +* Tr*cla4* were incubated on an SDA plate with or without 32 ng/mL itraconazole (Itra) or 3.0 ng/mL terbinafine (Terb) for 10 days (G). The diameter of the mycelium was measured (H). The dots on the graph represent the diameter of individual samples. *n* = 3 each.

Considering the conserved role of PAKs in promoting actin polymerization (26 – 28), we examined actin localization in the ΔTr*cla4* strain. Actin accumulation at the hyphal tip was attenuated in ΔTr*cla4* compared to the WT (Fig. 4A and B; Fig. S2B). The expression level of the actin gene (*ACTB*) was comparable among WT, ΔTr*cla4*, and ΔTr*cla4* + Tr*cla4* (Fig. 4C). The protein level of actin in ΔTr*cla4* was also similar to that in WT when equal amounts of total protein were loaded (Fig. 4D). These results suggest that TrCla4 plays a role in regulating mycelial growth, hyphal morphogenesis, and actin localization. To investigate whether TrCla4 promotes mycelial growth through mechanisms other than actin polymerization, we treated WT, ΔTr*cla4*, and ΔTr*cla4* + Tr*cla4* with cytochalasin A (Cyto A). Mycelial growth in WT and ΔTr*cla4* + Tr*cla4* was inhibited by Cyto A, while its inhibitory effect on mycelial growth in ΔTr*cla4* was not additive (Fig. 4E and F). In contrast to Cyto A, itraconazole and terbinafine, the inhibitors of the ergosterol biosynthesis pathway, which is involved in hyphal elongation through a pathway distinct from actin polymerization, exhibited additive effects on mycelial growth in ΔTr*cla4*, showing similar growth inhibition as Cyto A (Fig. 4G and H). These results suggest that TrCla4 promotes mycelial growth in *T. rubrum* primarily by facilitating actin polymerization.

### Cla4 inhibitor IPA-3 and FRAX486 inhibit mycelial growth and attenuate the pathogenic activity of *T. rubrum* in an invertebrate infection model

**Fig 5:**
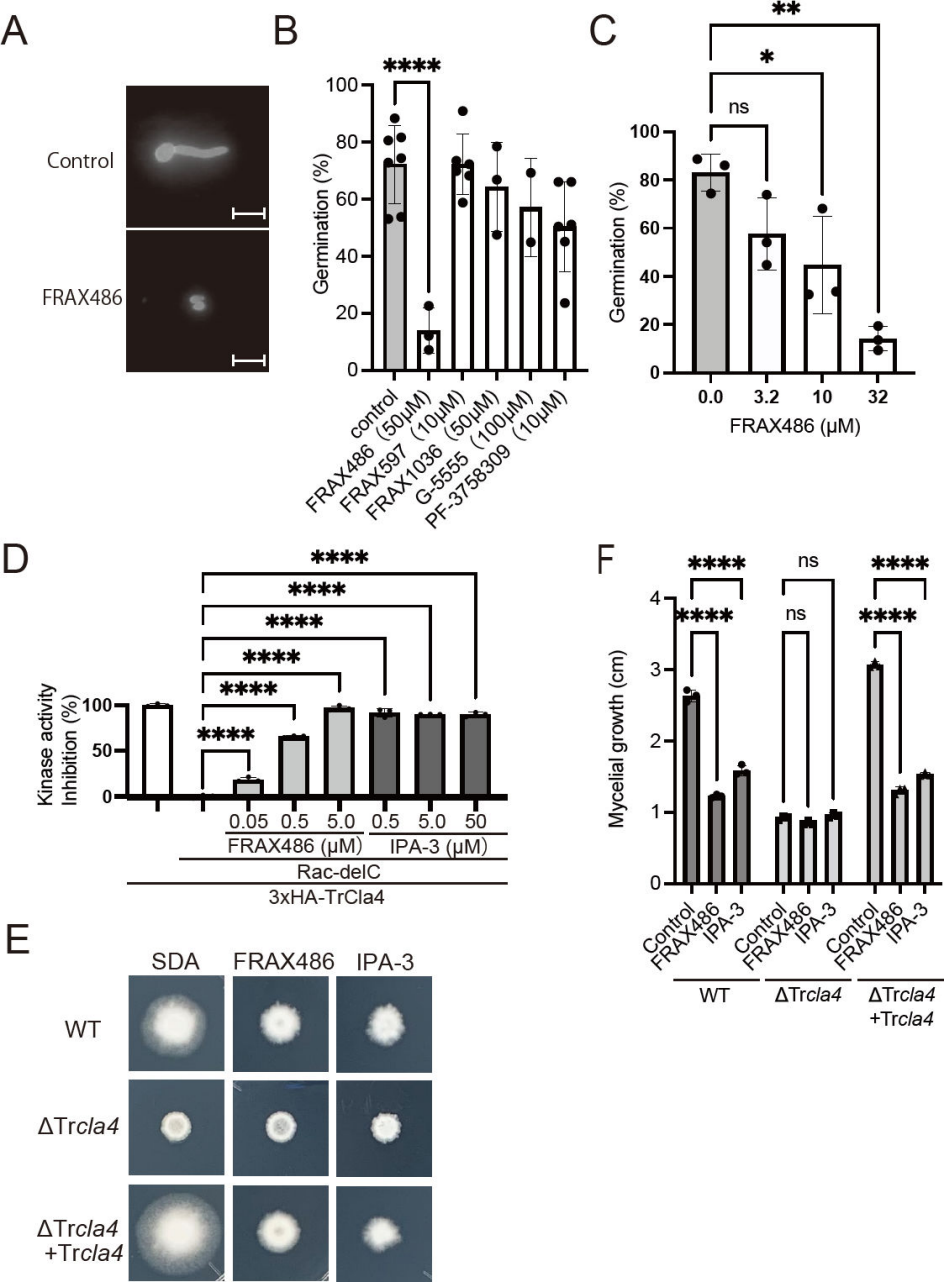
Sensitivity of WT and ΔTr*cla4* to PAK kinase inhibitors IPA-3 and FRAX486. (A) Spores of *T. rubrum* were incubated in SD medium with 1% DMSO (control) or FRAX486. Spores were stained with Calcofluor white, and spore germination was observed using fluorescent microscopy. (B) Spores of *T. rubrum* were incubated in SD medium with 1% DMSO (control), 50 µM FRAX1036, 50 µM FRAX486, 100 µM G-5555, 10 µM FRAX597, or 10 µM PF-3758309 at 28°C for 21 h (*n* = 7 control, *n* = 3 FRAX1036 and FRAX486, *n* = 2 G-5555, and *n* = 6 FRAX597 and PF-3758309). Germinated and ungerminated spores were counted, and the ratio of germinated spores was calculated. The dots on the graph represent the germination ratio of individual samples. (C) Spores of *T. rubrum* were incubated in SD medium with 0, 3.2, 10, and 32 µM FRAX486 at 28°C for 21 h. Germinated and ungerminated spores were counted, and the ratio of germinated spores was calculated. The dots on the graph represent the germination ratio of individual samples (*n* = 3 each). (D) Rac-dependent kinase activity of immunoprecipitated 3xHA-Cla4 was inhibited by 0.05–5.0 µM FRAX486 and 0.5–50 µM IPA-3 (*n* = 3 each). (E and F) Spores of WT, ΔTr*cla4*, and ΔTr*cla4 +* Tr*cla4* were incubated on an SDA plate with or without 3.1 µM FRAX486 or 50 µM IPA-3 for 10 days (E). The diameter of the mycelium was measured (F) (*n* = 3 each). The dots on the graph represent the diameter of individual samples.

We examined five inhibitors of mammalian PAK kinase activity, including FRAX486, FRAX597, G-5555, and PF-3758309, to determine if PAK kinase activity was involved in the germination of *T. rubrum* (Fig. 5A and B). FRAX486 was one of these inhibitors, and it decreased *T. rubrum* germination in a dose-dependent manner (Fig. 5C). To evaluate the inhibitory effects of FRAX486 on TrRac-dependent Cla4 kinase activity, we conducted immunoprecipitation of 3xHA-tagged *Cla4* proteins and measured the ATP consumption of the fusion proteins in the presence of recombinant TrRac using the Kinase-Glo assay kit. Immunoprecipitated 3xHA-Cla4 led to ATP consumption upon the addition of recombinant TrRac (Fig. 5D). Both FRAX486 and IPA-3 inhibited the TrRac-dependent ATPase activity of 3xHA-TrCla4 (Fig. 5D). These findings suggest that FRAX486 inhibits fungal PAK kinase activity. Assuming that FRAX486 and IPA-3 target TrCla4 *in vivo*, we further examined the sensitivity of the ΔTr*cla4* strain to these inhibitors. FRAX486 also inhibited mycelial growth in the WT strain (Fig. 5E and F). At concentrations that significantly inhibited mycelial growth in the WT and ΔTr*cla4* + Tr*cla4* strains, FRAX486 and IPA-3 did not exhibit an additional inhibitory effect on mycelial growth in the ΔTr*cla4* strain (Fig. 5E and F). These results suggest that both FRAX486 and IPA-3 inhibit TrCla4 and that the CRIB domain (inhibited by IPA-3) and kinase activity (inhibited by FRAX486) of TrCla4 are crucial for mycelial growth in *T. rubrum*.

**Fig 6:**
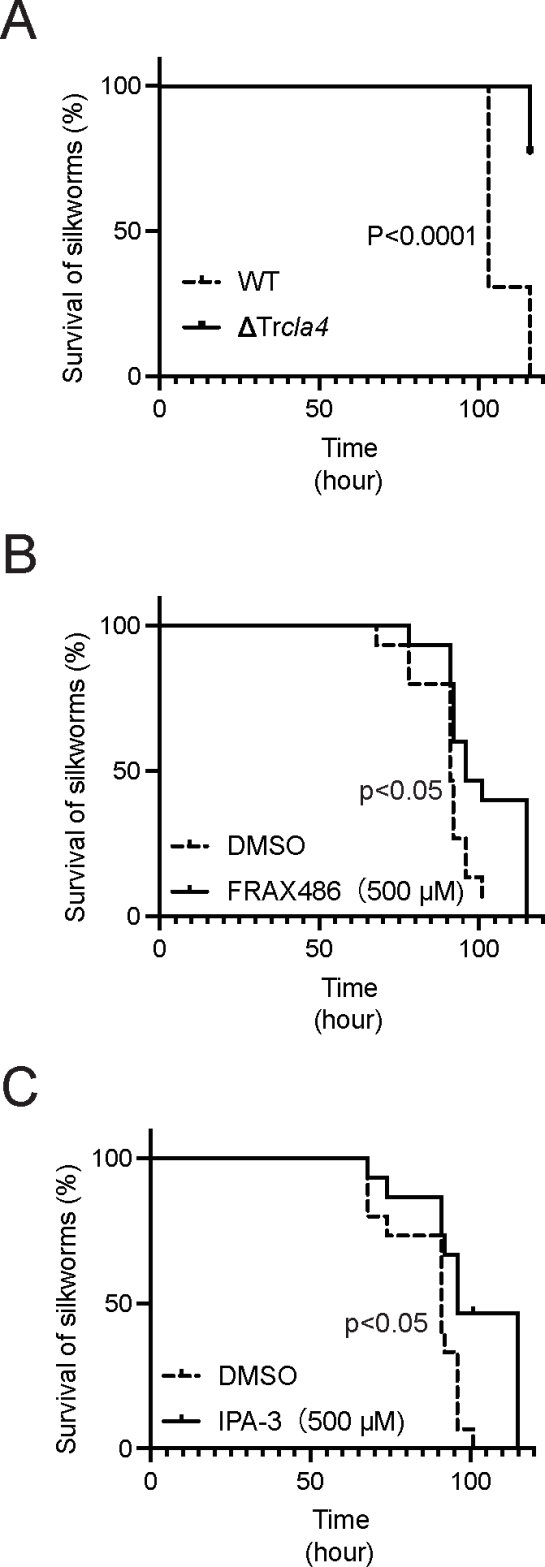
Amelioration of *T. rubrum*-infected silkworm survival by PAK kinase inhibitors IPA-3 and FRAX486. (A) Silkworms were injected with 0.5 × 10^6^ spores of *T. rubrum* WT or ΔTr*cla4*. Silkworms were reared at 30°, and their survival was monitored (*n* = 13 each). (B) Silkworms were injected with 1.3 × 10^6^ spores of *T. rubrum*, followed by an injection of 50 µL of the vehicle or 0.5 mM FRAX486. Silkworms were reared at 30°, and their survival was monitored (*n* = 15 each). (C) Silkworms were injected with 1.3 × 10^6^ spores of *T. rubrum*, followed by an injection of 50 µL of the vehicle or 0.5 mM IPA-3. Silkworms were reared at 30°, and their survival was monitored (*n* = 15 each).

To evaluate the TrCla4 as a potential therapeutic target, we utilized a silkworm infection model (29, 30). Injecting the ΔTr*cla4* strain into the silkworms resulted in a statistically significant increase in the survival time compared to the WT, suggesting that TrCla4 promotes pathogenicity against silkworms (Fig. 6A). Treatment with FRAX486 and IPA-3 led to a statistically significant improvement in silkworm survival (Fig. 6B and C). These findings suggest that TrCla4 could potentially serve as a therapeutic target for dermatophytosis.

## DISCUSSION

In the context of mycelial growth, the involvement of actin polymerization has been documented in various fungi (10 – 12) but remains unknown in the dermatophyte *T. rubrum*. In this study, we aimed to elucidate the significance of actin polymerization and the Rac/CDC42-PAK pathway in the mycelial growth of *T. rubrum*, with the objective of identifying a novel target for inhibitors against fungal growth. Our investigation revealed that TrCla4, a PAK protein in *T. rubrum,* enhances mycelial growth and actin polymeization *in vitro* as well as virulence against infected silkworms. Inhibitors of TrCla4, namely IPA-3 and FRAX486, hindered mycelial growth and improved the survival rate of *T. rubrum*-infected silkworms. These results suggest the potential of dermatophyte PAK as a therapeutic target for dermatophytosis.

The mechanism by which the TrCla4 protein is involved in actin polymerization is not yet known. One possible model is that TrCla4 phosphorylates type I myosin to activate the arp2/3 complex, thereby promoting actin polymerization at the hyphal tip. The following previous reports support this possibility: (i) In budding yeast, type I myosin promotes actin polymerization via the arp2/3 complex downstream of CDC42 (18). (ii) Type I myosin Myo3 is known to be phosphorylated at serine 357 by PAKs, which are direct effectors of CDC42 (17). (iii) Replacement of the serine 357 of type I myosin with aspartic acid, a phosphomimic mutation of serine 357, suppresses the impairment of actin polymerization caused by CDC42 deficiency (18). Furthermore, the 357th serine residue of type I myosin is also conserved in *T. rubrum* (TERG_12303, Fig. S3). Further studies are required to identify the phosphorylation substrates that lead to the promotion of actin polymerization downstream of TrCla4, as many substrates for PAK proteins other than type I myosin have been reported (19, 20).

The Cla4 inhibitor IPA-3 retarded germination of *T. rubrum* and no hyphal elongation was observed in Fig. 1C (incubated for 21 h), whereas ΔTr*cla4* showed hyphal elongation in Fig. 3F (incubated for 48 h). The difference in incubation time is likely to affect the presence or absence of hyphal elongation. Indeed, conidia treated with higher doses of IPA-3 showed mycelial growth, albeit at a reduced growth rate after prolonged incubation (Fig. 1E). These observations suggest that the function of TrCla4 is not to initiate but to promote germination. The increased branching in ΔTr*cla4* strains (Fig. 3G and H) also suggests that TrCal4 is not essential for initiating hyphal elongation but may be required to maintain the elongation point. In fungal phytopathogen, *Bipolaris maydis*, Cla4 has been found to be involved in maintaining the localization of the Spitzenkörper, which is thought to be a polarity regulator of mycelial elongation (31). Further studies are required to elucidate the detailed function of TrCla4.

While IPA-3 and FRAX486 have previously been shown to impede actin organization and cell proliferation in mammalian cell lines (32, 33), their inhibitory effects on mycelial growth and the function of fungal PAK proteins have not been reported. Griseofulvin, an antifungal drug traditionally used for the treatment of dermatophytosis, exerts its antifungal activity by inhibiting the polymerization of microtubules in the cytoskeleton. This suggests that the cytoskeleton and associated molecules may harbor untapped therapeutic targets beyond microtubules. Given that IPA-3 and FRAX486 target regulatory proteins involved in cytoskeleton polymerization, they hold promise as lead compounds for the development of novel therapeutic agents against dermatophytosis.

FRAX486 inhibited mycelial growth in *T. rubrum*. FRAX486 has higher inhibitory activity against group 1 PAKs than group 2 PAKs (34). TrCla4 is more homologous to mammalian group 1 PAKs than group 2 PAKs, which may explain why FRAX486 inhibits mycelial growth in *T. rubru*m. The other four inhibitors of PAK kinase activity used in this study (FRAX597, FRAX1036, G-5555, and PF3758309) did not inhibit mycelial growth. These compounds may have low transfer efficiency into the fungal cytoplasm, low stability in fungal cells, and/or low binding affinity for fungal PAKs. Although IPA-3 does not inhibit ΔTr*cla4* growth even at high concentrations, the ATP-competitive group I PAK inhibitor FRAX486 (35) inhibited ΔTr*cla4* growth at high concentrations (data not shown), possibly due to an off-target inhibitory effect. ATP-competitive inhibitors tend to show a broad inhibitory spectrum across kinases due to the conservative nature of the ATP binding pocket among kinases (36). Since Cla4 and Ste20, another PAK protein, are synthetically lethal in *Saccharomyces cerevisiae* (37), FRAX486 may also inhibit TrSte20 and lead to a synthetic effect in *T. rubrum*.

TrCla4 and TrSte20 were identified as potential PAK proteins in *T. rubrum* through a database search. It was observed that TrCla4 interacts with activated TrRac and promotes mycelial growth in this dermatophyte. However, the functional role of TrSte20 as a PAK in *T. rubrum* remains unknown. Attempts were made to generate a deletion mutant of Tr*ste20* by replacing its ORF with *nptII* using the ATMT method. However, no deletion mutant was successfully obtained. This suggests that there might be a preference for a specific genomic region in the ATMT method. Alternative approaches such as PEG-mediated transformation or the CRISPR/Cas9 system, which has been recently established, may prove helpful in generating the desired mutants (38).

In an invertebrate infection model, we discovered that fungus PAK inhibitors prevented infection aggravation. The kinase and CRIB domains of TrCla4 and human PAK1 had sequence identities of 54% and 59%, respectively, demonstrating the significant sequence similarity of PAK across species. PAK is also found in mammals, including humans. Because it shields keratinocytes from DNA damage and apoptosis brought on by UV-B and plays a role in epithelial wound healing, PAK1 is a crucial protein for maintaining the homeostasis of the human epidermis (39, 40). As a result, in order to create medications that are uniquely poisonous to fungus, fungal PAKs must be targeted for research. In the future, high-throughput searches for highly specific chemicals will call for the development of an evaluation system for fungal PAK activity as well as three-dimensional structural analysis to clarify the structural distinctions between the host and pathogen proteins.

This work is the first to our knowledge to show actin localization in dermatophyte cells. To see polymerized F-actin, we first tried phalloidin. However, we were unable to identify any distinct signals coming from the fungi. This could be as a result of the 180th amino acid substitution in *T. rubrum* actin, methionine for leucine, which has been linked to phalloidin binding (41). Actin was shown to be localized at the hyphal tip, and *T. rubrum*’s mycelial growth was inhibited by cytochalasin A. These findings suggest that mycelial proliferation in dermatophytes is similarly influenced by actin polymerization at the hyphal tip.

This research discovered FRAX486 as a fungus PAK kinase activity inhibitor and IPA-3 as an inhibitor of fungal p21-PAK interaction. These substances are anticipated to be used in the investigation of the molecular function of PAKs in other dermatophyte species as well as other pathogenic fungi, such as *Aspergillus* spp. and Mucorales. Future research will need the creation of instruments for the simple detection of fungal PAK kinase activity as well as the screening of analogs of IPA-3 and FRAX486 that function in a fungus-specific way.

## MATERIALS AND METHODS

### Fungal and bacterial strains and culture condition

*Escherichia coli* BL21 (Takara Bio, Inc., Japan), *A. tumefaciens* EAT105 (42), and *T. rubrum* CBS118892 (43), a clinical isolate from a patient’s nail, were cultured under the following conditions: *E. coli* BL21 was grown in LB medium with appropriate antibiotics at 37°C, *A. tumefaciens* EAT105 was cultured in LB or *Agrobacterium* induction medium supplemented with 0.2 mM acetosyringone at 28°C, and *T. rubrum* CBS118892 was cultivated on Sabouraud dextrose agar (SDA; 1% Bacto peptone, 4% glucose, and 1.5% agar, pH unadjusted) at 28°C. The conidia of *T. rubrum* were prepared as previously described (44). To assess the inhibitory effect of PAK inhibitors on mycelial growth, wild-type (WT), Tr*cla4* knockout (ΔTr*cla4*), and Tr*cla4* reconstituted (ΔTr*cla4 +* Tr*cla*) strains of *T. rubrum* were cultured on modified SDA, where agar was substituted with agarose.

### Inhibitors

Cayman Chemical Company (USA) provided IPA-3, FRAX486, FRAX1036, FRAX597, PF-3758309, G-5555, and cytochalasin A. Tokyo Chemical Industry Co., Ltd. (Japan) supplied the itraconazole and terbinafine. Dimethyl sulfoxide (DMSO) was used to dissolve the inhibitors. Using Prism 9 (GraphPad, USA), the half-maximum inhibitory concentration (IC_50_) was calculated.

### Germination assay

Fungal conidia (2 × 10^6^) were incubated in Sabouraud dextrose medium (SD medium; 1% Bacto peptone and 4% glucose) with 1% DMSO (control) or the indicated concentration of PAK inhibitors for 21 h, washed two times with saline, stained with 0.2 mM Calcofluor white (Sigma, USA), and observed using fluorescent microscopy (BX53, Olympus, Japan) and a VGA camera (MS-200, BIO CRAFT, Japan). If the protrusion’s length was greater than the conidia’s diameter, the conidia were regarded as having germinated.

### Plasmid construction

Tr*Rac* was amplified using PCR and then inserted into the EcoRI-BamHI sites of the pGEX6p-1 plasmid (Global Life Sciences Technologies Japan K.K., Japan). The identification of TrCla4 and Tr*Ste20* was performed by searching the respective sequences in a database using the basic local alignment search tool (BLAST, available at https://blast.ncbi.nlm.nih.gov/Blast.cgi). 6× His-, Tf-, and FLAG-tagged versions of TrCla4 and Tr*Ste20* were amplified using PCR and cloned into the pCold I DNA plasmid (Takara Bio, Inc.) with the In-Fusion system (Takara Bio, Inc.).

A Tr*cla4*-targeting vector, pAg1-ΔTr*cla4*, was constructed using the following procedure: First, approximately 1.8 and 1.9 kb fragments of the 5′ and 3′-UTR regions of the Tr*cla4* open reading frame (ORF) were amplified from *T. rubrum* genomic DNA through PCR. The plasmid backbone of pAg and the antibiotic resistance gene cassette were obtained by PCR amplification from the pAg1-3′-UTR of ARB_02021. Finally, these four fragments were fused together using the In-Fusion system (Takara Bio, Inc.).

To construct a vector for 3×HA-TrCla4 overexpression, pAg1-hph-3xHA-Cla4, the following steps were performed: First, the antibiotic resistance gene cassette (hph) was amplified via PCR. Subsequently, the *tef1* promoter (Ptef1) was amplified from the *T. rubrum* genome by PCR. The 3×HA tag was amplified from the vector 3×HA-TurboID-NLS_pCDNA3 (Addgene) by PCR. The Tr*cla4* gene was amplified from *T. rubrum* cDNA by PCR. Additionally, the plasmid backbone of pAg was amplified from the pAg 1–3′-UTR of ARB_02021 via PCR. Finally, these five fragments were joined together using the In-Fusion system (Takara Bio, Inc.).

### Preparation of TrRac

TrRac proteins were expressed in *E. coli* BL21 cells transformed with the pGEX6p-1 Tr*rac* plasmid. The transformed *E. coli* BL21 cells were pre-cultured in 5 mL of LB medium containing 50 µg/mL ampicillin at 37°C. They were then inoculated into 2 L of LB medium supplemented with 50 µg/mL ampicillin and cultured at 37°C for 3 h. Subsequently, 0.1 mM isopropyl β-D-1-thiogalactopyranoside (IPTG) was added, and the cells were further incubated at 20°C overnight for protein induction. GST fusion proteins were purified using GSTrap HP (GE Healthcare) and purification buffer A (20 mM Tris-HCl pH 7.5, 150 mM NaCl, 2.5 mM MgCl_2_, and 0.5 mM dithiothreitol [DTT]), following the manufacturer’s instructions. The GST tag was removed by incubating the purified GST-TrRac protein with PreScission Protease at 4°C, and the flow-through fraction of the GSTrap column was collected. Desalting of TrRac was performed using an Amicon Ultra 10k centrifugal filter device (Merck, Germany), and the flow-through fraction of Q Sepharose HP (GE Healthcare) was collected. To bind guanine nucleotides to TrRac, purified TrRac (10 µg) was incubated with 100 µM GTPγS or GDP and 5 mM EDTA in buffer A at 28°C for 30 min. The reaction was stopped by adding MgCl_2_ to a final concentration of 30 mM.

### Pull-down assay

TrCla4 and TrSte20, tagged with His-, Tf-, and Flag-tags at their N-termini, were expressed following the instruction manual of the pCold vector (Takara Bio, Inc.). The precipitate of BL21 expressing Tf-TrCla4 and Tf-TrSte20 was stored at −25°C until use. Prior to use, the *E. coli* precipitate was suspended in 1 mL of buffer A. After centrifugation at 4°C and 15,000 rpm for 5 min, the supernatant was supplemented with a final concentration of 30 mM MgCl2. The prepared lysate supernatants of TrCla4 and TrSte20 were then mixed with purified TrRac bound with GTPγS or GDP, and 20 µL of equilibrated Ni NTA agarose beads (50% suspension, Merck). The mixture was incubated at 4°C for 2 h. For the binding inhibition assay with IPA-3, either 1% DMSO or a final concentration of 0.32, 1.0, 3.2, or 10 µmol/L of IPA-3 was added to the reaction. After incubation, the beads were washed three times with buffer A supplemented with 30 mM MgCl_2_ and 10 mM imidazole, followed by an additional wash with buffer A containing 30 mM MgCl_2_. Samples were eluted with 2 × Laemmli’s sample buffer and incubated at 95°C for 5 min. The centrifuged supernatant was then applied to SDS-PAGE and stained with Coomassie Brilliant Blue (CBB) to visualize the TrRac band.

### Transformation of *T. rubrum*

The *A. tumefaciens*-mediated transformation (ATMT) technique was used to alter *T. rubrum*, as previously mentioned (4, 45, 46). PCR was used to assess the intended transformants and pure genomic DNA. The Quick-DNA Fungal/Bacterial Miniprep Kit (Zymo Research, USA) was used to extract the total DNA. T-01 (TAITEC, Japan) used 5 mm stainless steel beads to perform a study on the collision of beads with fungus cells.

### Quantitative reverse-transcription PCR (qPCR)

Total RNAs were purified using the NucleoSpin RNA kit (Macherey-Nagel, Germany) and then reverse-transcribed into cDNAs using the ReverTra Ace kit (Toyobo, Japan), following the manufacturer’s instructions. For qPCR analysis, TB Green Premix Ex Taq II (Takara Bio Inc.) was used on a StepOne real-time PCR instrument (Thermo Fisher Scientific, USA). The relative mRNA expression levels were determined using the 2^−∆∆Ct^ method, with *DNA-dependent RNA polymerase II* (*rpb2*) serving as the endogenous control for sample normalization (47). The primers utilized in this study are listed in Table S1.

### Western blotting

Conidia from *T. rubrum* were incubated in an SD medium for 3 days. Samples were harvested using Miracloth (Merck Millipore, USA) and immediately frozen in liquid nitrogen. Crushed mycelium weighing between 90 and 190 mg was obtained by running the Bead crusher (TAITEC, Japan) at 4200 rpm for 30 s. For protein extraction, 0.3 mL of lysis buffer (10% [vol/vol] glycerol, 50 mM Tris-HCl pH 7.5, 1% [vol/vol] Triton X-100, 150 mM NaCl, 20 mM EDTA, and Protease Inhibitor Cocktail [EDTA-free] [Nacalai tesque, Japan]) was added to the crushed mycelium and mixed using the Bead crusher. After incubating on ice for 10 min, the extracts were centrifuged at 15,000 × *g* for 5 min at 4°C. The supernatants were collected, and the protein concentrations were determined using the Bradford Assay. For SDS-PAGE, 12.5 µg of protein from each sample was separated and transferred to polyvinylidene difluoride (PVDF) membranes (Merck Millipore, USA). Actin was probed using an anti-actin antibody (clone C-4, Merck; diluted 1/1,000). Primary antibodies were detected using a horseradish peroxidase (HRP)-conjugated anti-mouse IgG antibody (Cell Signaling Technology, USA).

### Indirect immunofluorescence

WT, ΔTr*cla4*, or ΔTr*cla4 +* Tr*cla4* strains were seeded with 1–5 × 10^6^ spores on sterile cover glasses placed in a 12-well plate. They were then incubated with 500 µL of SD liquid medium at 28°C overnight. On the second day, the SD medium was replaced with fresh medium, and the spores were further incubated at 28°C overnight. On the third day, the supernatant was removed, and the cells were fixed with 4% paraformaldehyde (PFA; Nacalai Tesque, Japan) at room temperature for 15 min. In the case of testing cytochalasin A, 16 µM cytochalasin A was added to the medium 1 h before fixation. The samples were washed three times with PBST (PBS + 0.05% Tween 20) and then incubated in 400 µL of 10 mg/mL Lysing enzyme/10% bovine serum albumin (BSA)/PBS for 2 h at 28°C. Afterward, they were washed three times with PBST, permeabilized with 400 µL of pre-cooled methanol at −20°C for 10 min, and washed again with PBST. Next, the samples were incubated for 30 min with the blocking buffer (10% Donkey serum/0.2% Triton X-100/0.02% sodium azide/PBS), followed by incubation with anti-beta-actin antibody (clone C-4, Merck; diluted 1/1,000) in Can Get Signal A solution (Toyobo, Japan) at 4°C overnight. After washing three times with PBST, the samples were incubated with anti-mouse IgG antibody conjugated with Alexa488 (Abcam; diluted 1/1,000) and DAPI solution (Dojindo, Japan; diluted 1/100,000) in Can Get Signal A solution for 1 h at room temperature. They were then washed three times with PBST, rinsed with water, and mounted on glass slides using Aqua-Poly/Mount (Polysciences, UK). The stained cells were observed using a BZ-8100 all-in-one fluorescence microscope (Keyence, Japan) or a confocal microscope system AX (Nikon). To calculate relative fluorescence intensity, each strain was immunostained under identical conditions and fluorescence micrographs were taken using the same exposure time and gain settings. Relative fluorescence intensity at the hyphal tip was measured using ImageJ software (National Institutes of Health) and calculated by dividing the fluorescent intensity at the hyphal tip by the value of the hypha (10 µm proximal site from the hyphal tip).

### Phylogenic tree analysis

The neighbor-joining technique was used to estimate the evolutionary tree of fungal PAKs. The ideal tree is shown. The branches are accompanied by the percentage of duplicate trees in which the linked taxa were grouped during the bootstrap test (1,000 repetitions). With branch lengths in the same units as the evolutionary distances used to estimate the phylogenetic tree, the tree is rendered to scale. The evolutionary distances, which are measured in the number of amino acid substitutions per site, were calculated using the Poisson correction technique. There were 25 amino acid sequences in this investigation. With the complete deletion option, all places with gaps and incomplete data were removed. The final data set had 353 locations altogether. In MEGA11, evolutionary analyses were carried out.

### Immunoprecipitation

3×HA-TrCla4-overexpressed *T. rubrum* was cultured in SD medium at 28°C for 4 days. The cells were then washed with sterile water and immediately frozen in liquid nitrogen. To extract proteins, the frozen cells were transferred to a 2-mL screwcap tube containing 5 mm stainless steel beads and crushed using a Bead crusher (TAITEC) at 4,200 rpm for 30 s. For protein extraction, 0.5 mL of lysis buffer was added to the crushed mycelium and mixed using the Bead crusher. After incubating on ice for 10 min, the extracts were centrifuged at 15,000 × *g* for 5 min at 4°C. The resulting extracts were incubated with 10 µg of anti-HA antibody (3F10, Roche) and 25 µL of Protein L Magnetic Beads (Pierce), according to the manufacturer’s protocol, at 4°C for 2 h. Unbound material was removed following the manufacturer’s protocol with lysis buffer. The beads were then resuspended in 200 µL of kinase buffer (50 mM Tris/HCl pH 7.5, 20 mM MgCl2, and 0.1% BSA) and used immediately for kinase assays.

### Kinase assays

Kinase assays were conducted using the Kinase-Glo assay kit (Promega). The assays were performed in kinase buffer supplemented with 10 µM ATP, 0.1 mg/mL myelin basic protein bovine (Sigma-Aldrich), and 5 µL of 3×HA-TrCla4-conjugated magnetic beads and/or purified recombinant Rac-delC (2 µg/reaction). The protein kinase inhibitors IPA-3 and FRAX486 were included where specified. The kinase assays were carried out in 25 µL reaction volumes for a duration of 30 min at 30°C. Luminescence was measured using a TriStar2 LB942 instrument (Berthold Technologies) in 384-well plates.

### Infection experiment using silkworms

Eggs from silkworms were purchased from Ehime-Sansh Co., Ltd. (Japan) and reared according to the previously reported method (48, 49). Fifth instar silkworm larvae were fed an artificial diet (Silkmate 2S; Ehime-Sanshu Co., Ltd., Japan) overnight (50). *T. rubrum* spores were suspended in 0.9% (wt/vol) NaCl. A suspension of fungal cells (50 µL) was injected into the silkworm hemolymph using a 1-mL tuberculin syringe (Terumo Medical Corporation, Tokyo, Japan). Immediately after injection, the silkworm hemolymph was further injected with either the vehicle (5% DMSO, 5% Tween 80, and 0.9% [wt/vol] NaCl), 0.5 mM FRAX 486, or 0.5 mM IPA-3 (50 µL). The injected silkworms were then placed in an incubator at 30°C, and their survival was monitored.

### Statistical analysis

The means of the two groups were compared using the Student’s *t* test. For three or more groups with a single variable, a one-way analysis of variance (ANOVA) with Tukey’s post hoc test was conducted. For means of three or more groups with two variables, a two-way ANOVA with Tukey’s post hoc test was performed. Prism 9 software (GraphPad) was utilized for these statistical analyses. Statistical significance was defined as *P* < 0.05.

## SUPPLEMENTAL MATERIAL

Fig. S1 spectrum.02923-23-s0001.tif Rhodamine phalloidin staining of hyphal tip of *T. rubrum*.

Fig. S2 spectrum.02923-23-s0002.tif Enhanced images of hyphae which were stained with anti-actin antibody and Alexa 488-conjugated anti-mouse IgG secondary antibody.

Fig. S3 spectrum.02923-23-s0003.tif The 357th serine residue of type I myosin Myo3 in S. cerevisiae is also conserved in diverse fungal pathogens.

Table S1 spectrum.02923-23-s0004.xlsx Primers used in this study.

ASM does not own the copyrights to Supplemental Material that may be linked to, or accessed through, an article. The authors have granted ASM a non-exclusive, world-wide license to publish the Supplemental Material files. Please contact the corresponding author directly for reuse.
