# Supplementary figures and images for: TrCla4 promotes actin polymerization at the hyphal tip and mycelial growth in Trichophyton rubrum
Source: Microbiol Spectr. 2023 Oct 31;11(6):e02923-23. doi: 10.1128/spectrum.02923-23 (PMC10714743; doi:10.1128/spectrum.02923-23)

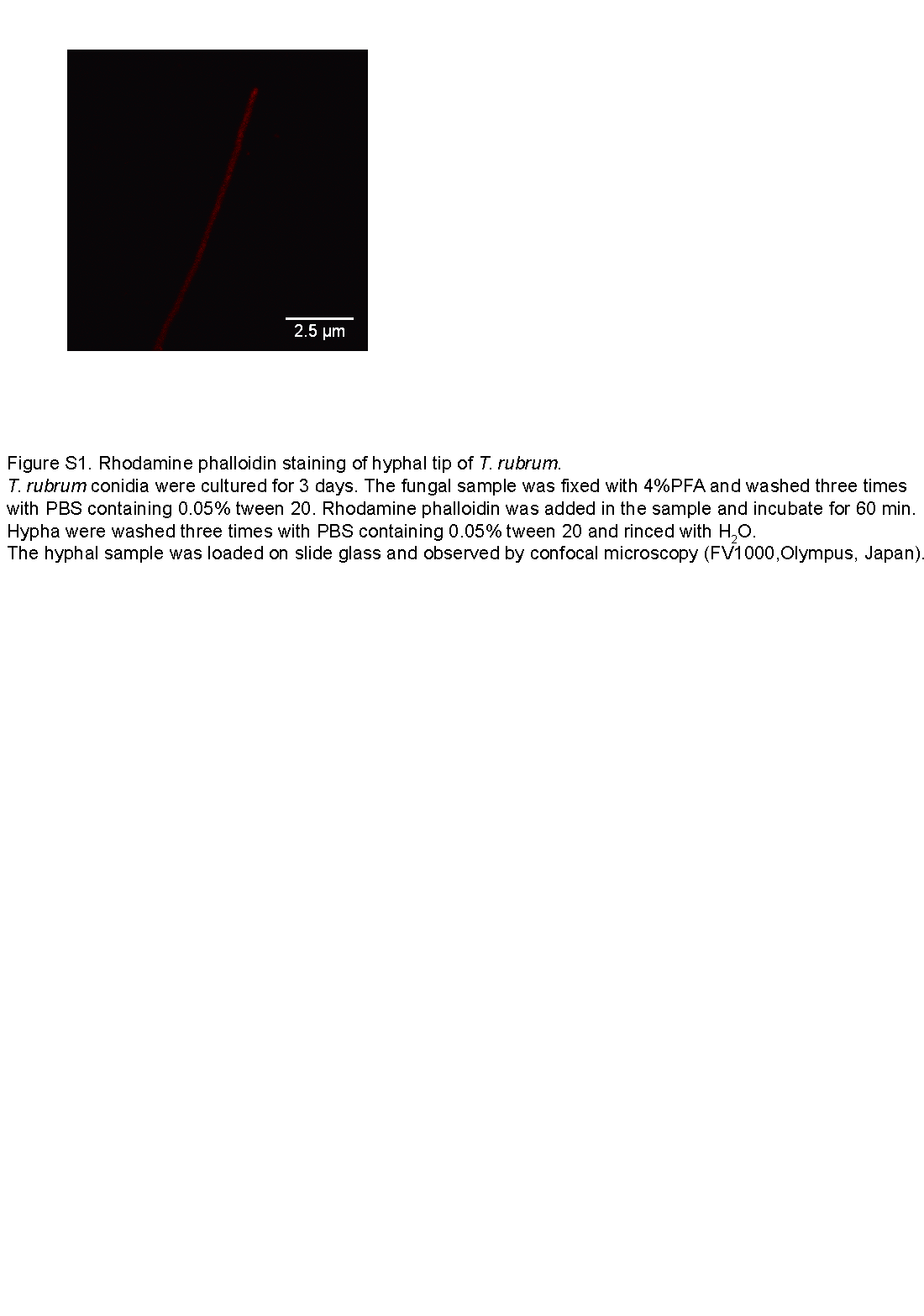

Supplement: Fig. S1 — Rhodamine phalloidin staining of hyphal tip of T. rubrum. [file spectrum.02923-23-s0001.tif]

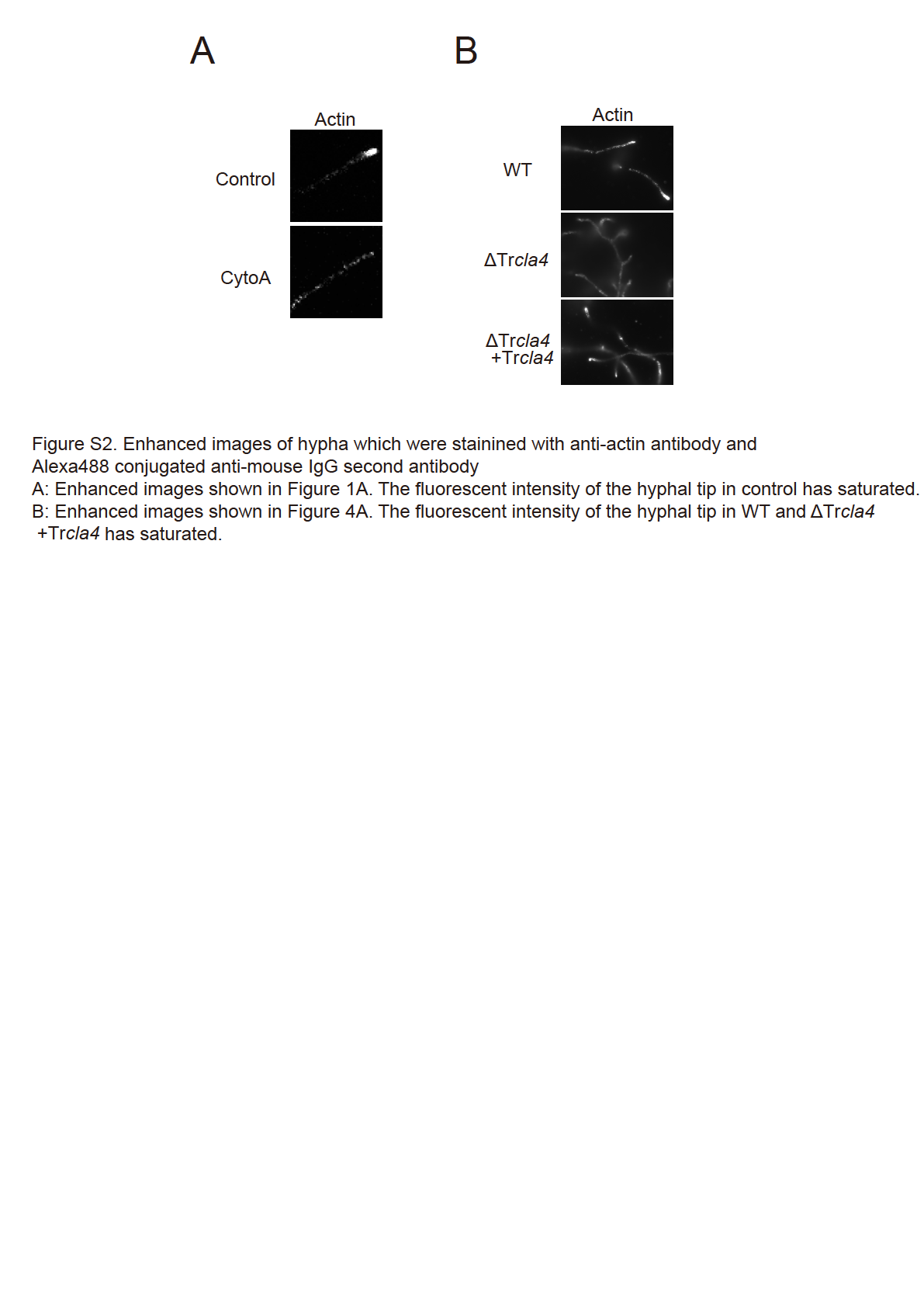

Supplement: Fig. S2 — Enhanced images of hyphae which were stained with anti-actin antibody and Alexa 488-conjugated anti-mouse IgG secondary antibody. [file spectrum.02923-23-s0002.tif]

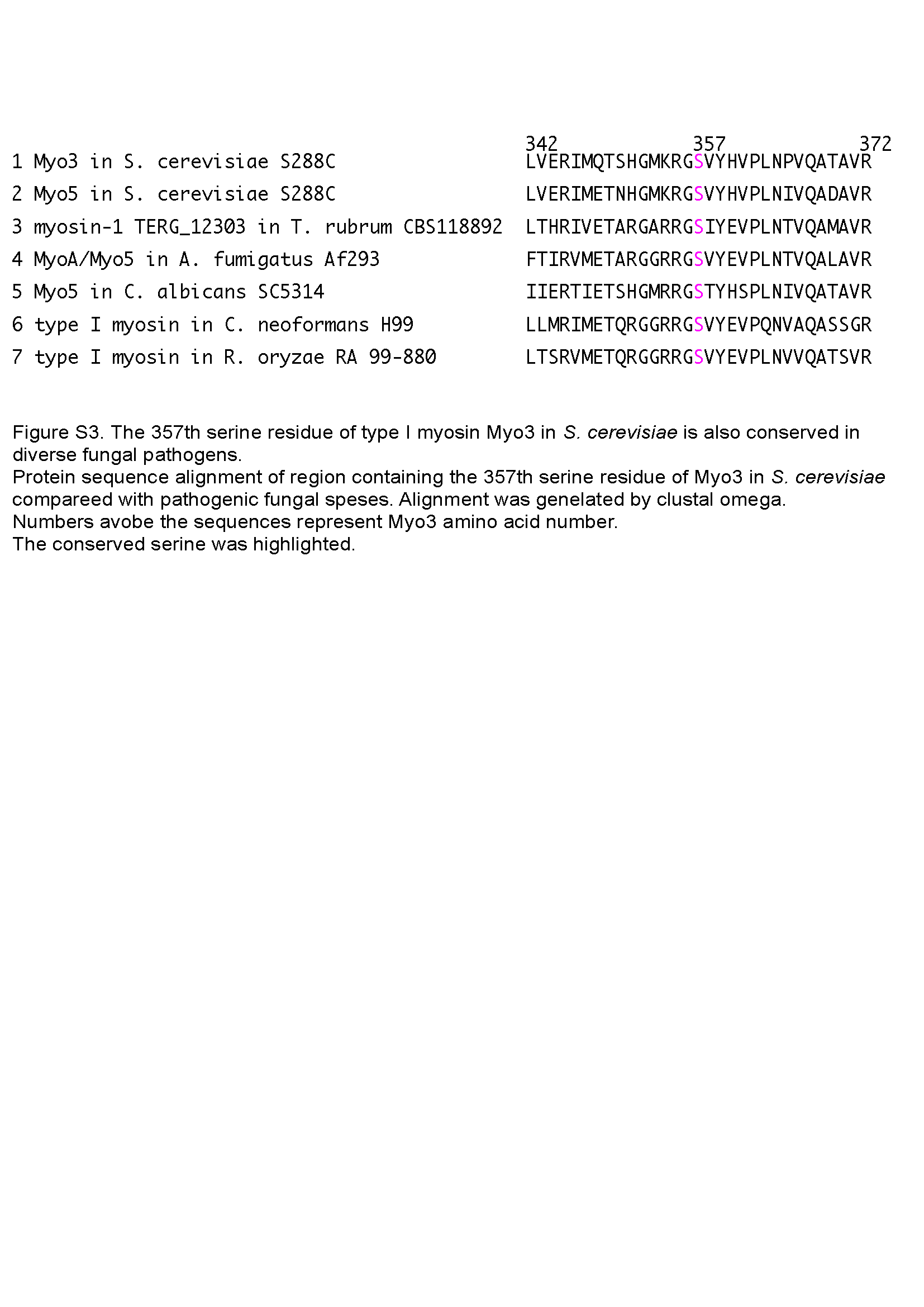

Supplement: Fig. S3 — The 357th serine residue of type I myosin Myo3 in S. cerevisiae is also conserved in diverse fungal pathogens. [file spectrum.02923-23-s0003.tif]
